# Blood Levels of Glial Fibrillary Acidic Protein (GFAP) in Patients with Neurological Diseases

**DOI:** 10.1371/journal.pone.0062101

**Published:** 2013-04-23

**Authors:** Christoph A. Mayer, Robert Brunkhorst, Marion Niessner, Waltraud Pfeilschifter, Helmuth Steinmetz, Christian Foerch

**Affiliations:** 1 Department of Neurology, Goethe-University, Frankfurt am Main, Germany; 2 Roche Diagnostics, Penzberg, Germany; Julius-Maximilians-Universität Würzburg, Germany

## Abstract

**Background and Purpose:**

The brain-specific astroglial protein GFAP is a blood biomarker candidate indicative of intracerebral hemorrhage in patients with symptoms suspicious of acute stroke. Comparably little, however, is known about GFAP release in other neurological disorders. In order to identify potential “specificity gaps” of a future GFAP test used to diagnose intracerebral hemorrhage, we measured GFAP in the blood of a large and rather unselected collective of patients with neurological diseases.

**Methods:**

Within a one-year period, we randomly selected in-patients of our university hospital for study inclusion. Patients with ischemic stroke, transient ischemic attack and intracerebral hemorrhage were excluded. Primary endpoint was the ICD-10 coded diagnosis reached at discharge. During hospital stay, blood was collected, and GFAP plasma levels were determined using an advanced prototype immunoassay at Roche Diagnostics.

**Results:**

A total of 331 patients were included, covering a broad spectrum of neurological diseases. GFAP levels were low in the vast majority of patients, with 98.5% of cases lying below the cut-off that was previously defined for the differentiation of intracerebral hemorrhage and ischemic stroke. No diagnosis or group of diagnoses was identified that showed consistently increased GFAP values. No association with age and sex was found.

**Conclusion:**

Most acute and chronic neurological diseases, including typical stroke mimics, are not associated with detectable GFAP levels in the bloodstream. Our findings underline the hypothesis that rapid astroglial destruction as in acute intracerebral hemorrhage is mandatory for GFAP increase. A future GFAP blood test applied to identify patients with intracerebral hemorrhage is likely to have a high specificity.

## Introduction

Recently, the astroglial protein GFAP has been identified as a potential blood biomarker of intracerebral hemorrhage (ICH) in patients with symptoms of acute stroke. [1]–[4] GFAP is released rapidly in case of an expanding parenchymal bleeding in the brain leading to immediate cell destruction, whereas it is detected with delay in case of ischemic stroke (IS), where necrosis and cellular disintegration do not occur before 6–12 h after symptom onset. The different kinetics of GFAP release in ICH and IS, respectively, may allow for an early, sensitive and specific distinction between the two major entities of stroke, potentially facilitating the triage of patients and fostering super early implementation of individually targeted therapies [4].

GFAP is the main intermediary filament of astrocytes, the most abundant cell type in the human central nervous system (CNS). [5], [6] It plays an essential role in maintaining shape and motility of astrocytic processes and contributes to white matter architecture, myelination and blood-brain barrier integrity. GFAP was found to be highly brain specific, as relevant extracerebral sources of this protein have not been identified. As a consequence, blood levels of GFAP in healthy individuals are very low, typically not exceeding the lower detection limits of the used tests. [4], [7], [8] Essential for the release of GFAP from brain tissue into the blood stream is supposed to be (I) the loss of astrocytic structural integrity due to necrosis and/or mechanical disruption and (II) disintegration of the blood-brain barrier. [1] It is not yet clear, however, whether the upregulation of GFAP following different pathological events in the CNS, a process commonly known as reactive astrogliosis, may lead to GFAP release and emergence of detectable protein levels in peripheral blood [9].

Apart from a considerable number of acute stroke patients, whose GFAP blood levels are reported in the literature, only little is known about GFAP in patients with other neurological diseases. GFAP was found elevated in the plasma of patients with glioblastoma, whereas other intracranial tumors including metastases were not shown to increase GFAP blood levels. [7], [10], [11] Furthermore, GFAP blood levels were shown to correlate with severity and outcome after traumatic brain injury. [12]–[14] Sporadically it was reported that patients with neuromyelitis optica show increased GFAP plasma levels [15].

In view of the potential use of GFAP as a diagnostic marker of acute ICH, the present investigation aims at providing a cursory overview of GFAP levels in a broad and rather unselected spectrum of neurological conditions and diseases, other than stroke, brain tumor and traumatic brain injury. We will provide data on individual GFAP levels and discuss the diagnostic value of GFAP testing in view of potential “specificity gaps”.

## Methods

### Ethics Statement

The study protocol was approved by the ethics committee of the Goethe-University, Frankfurt am Main, Germany.

### Patient Inclusion and Blood Sampling

Within a one-year period (May 2010 to April 2011), three inpatients of our university hospital were randomly selected on every working day (Monday to Friday) as potential study candidates from all patients who had undergone blood withdrawal for routine laboratory testing in the morning of that day. Patients below 18 years of age as well as patients diagnosed as having IS, transient ischemic attack, ICH, traumatic brain injury or brain tumor were excluded from the analysis (due to the already existing data from prospective GFAP studies concerning these disorders). Candidates or legal representatives were informed about the purpose of the study and written informed consent was obtained. 1 ml of EDTA-plasma was diverted from the blood drawn earlier that day for routine purposes and transferred into an Eppendorf tube. Within 60 minutes after blood draw, samples werde centrifugated at 10,000 g for 4 min, and the supernatant was immediately frozen and stored at −25°C. To assure specimen stability, shipment of samples was performed on dry ice in order to maintain the cool chain. GFAP is known to be stable in whole blood for several days at 4°C, and freezing and thawing for up to four cycles was found to not influence GFAP concentrations [8].

### Clinical Parameters

Primary endpoint was the final diagnosis reached at hospital discharge based on all available clinical and laboratory findings, including brain imaging. Diagnoses were classified according to ICD-10. In addition, the following clinical data were prospectively collected: age, sex and comorbidities such as concomitant neoplastic disease, concomitant cardiovascular disease, arterial hypertension, and diabetes mellitus.

### GFAP Determination

All scientists involved in the GFAP measurements were fully blinded to clinical data. Determination of the GFAP plasma concentrations was performed at Roche Diagnostics, Penzberg, Germany. An electrochemiluminometric immunoassay for the *in vitro* quantitative determination of GFAP in human serum and plasma (Elecsys® GFAP prototype test) was used. [4] In a first step, biotin- and ruthenium-labelled monoclonal GFAP antibodies were combined with 50 µl of sample and incubated for 9 min. In the second step, streptavidin-coated magnetic microparticles were added, and the mixture was incubated for 9 min. Then, the reaction mixture was transferred into the measuring cell where the beads were magnetically captured on an electrode surface. Unbound label was removed by washing the measuring cell. In the last step, voltage was applied to the electrode in the presence of a tri-propylamine (TPA)-containing buffer and the resulting electrochemiluminescent signal was recorded by a photomultiplier.

The GFAP concentration was calculated from the read-off on the basis of a standard calibration curve, defined by a set of seven master calibrators. Since an acknowledged reference method for GFAP is lacking to this day, the Elecsys® GFAP assay was standardized by weighing pure human GFAP in an analyte-free human serum matrix.

Each GFAP measurement was performed in full calibration mode. The measuring range of the GFAP prototype assay is between 0.05 and 150 µg/l (defined by the lower detection limit and the maximum of the master curve). The lower detection limit of the assay, defined as the lowest measurable GFAP concentration distinguishable from zero, was calculated as the concentration two standard deviations above the lowest standard at 0.05 µg/l. Intraassay precision was determined using 4 individual samples run in 21 replicates in a single run and found to range between 1.1% and 1.9%. Interassay precision was determined using the same samples run in duplicates in 10 individually calibrated runs and ranged between 2.7–4.2%.

In a collective of 132 apparently healthy individuals, GFAP plasma levels were determined to be 0.07±0.11 µg/l using this assay. The determination procedure was identical to that used in the BEFAST trial [4].

### Statistical Analysis

GFAP data did not follow a normal distribution. Thus, non-parametric statistics including median/interquartile range were used for data exploration, but mean±SD values were also calculated. Correlation analysis was performed using the Spearman rank test. Mann-Whitney U test was used to compare differences between two groups. Differences between all diagnosis groups were compared by means of the Kruskal-Wallis test and Dunn’s correction for multiple testing.

## Results

**Figure 1:**
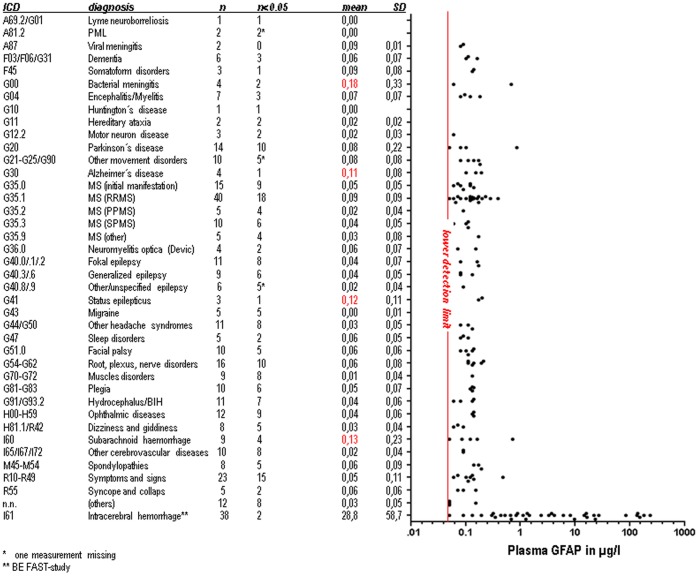
Patients are grouped according to discharge diagnosis (“ICD” = International Classification of Diseases). “n” depicts the number of patients per diagnosis. “n<0.05” displays the number of patients per diagnosis with GFAP values below the lower detection limit of the used immunoassay. The remaining values (those above the lower detection limit) are displayed as individual values in the graph. Individual patients with increased GFAP values are easy to recognize. Mean GFAP values and standard deviation (SD) are also provided for each diagnosis. The diagnoses with the three highest mean GFAP values are labelled in red. * = GFAP value of one sample is missing. ** = Results of the BE FAST study for comparison [4].

A total of 331 patients were included in the study. Mean age was 51±18 years, and 54% were female. Patients had a broad spectrum of discharge diagnoses, basically covering all relevant fields of neurological diseases (apart from IS, ICH and brain tumors; see Figure 1). Concerning non-neurological comorbidities, 6% of the patients had a co-existing neoplastic disease, 14% had a history of cardiovascular diseases, 20% had arterial hypertension and 11% diabetes mellitus.

Calculated for the entire dataset, the mean GFAP plasma level was 0.06±0.10 µg/l (median 0.00, interquartile range 0.00–0.10, minimum 0.00, maximum 0.85). 62.5% of GFAP values were found below the detection limit of the applied GFAP immunoassay (0.05 µg/l), whereas 98.5% of GFAP values lay below the cut-off of 0.29 µg/l that was previously identified as a good discriminator between ICH and IS. GFAP values were found to be unaffected by gender and evenly distributed between male and female patients. GFAP levels did not increase with age. No influence was found for GFAP regarding the above listed comorbidities.

No disease category could be identified with consistently increased GFAP values, as is the case in acute ICH (see Figure 1; Kruskal Wallis-test, p = 0.190). The three diagnostic groups displaying the highest GFAP mean values were bacterial meningitis (0.18±0.33 µg/l), subarachnoid hemorrhage (0.13±0.23 µg/l), and status epilepticus (0.12±0.11 µg/l). The three highest individual GFAP values were found in a case of Parkinson’s disease (0.85 µg/l), in a patient with subarachnoid hemorrhage (0.71 µg/l), and in a case of bacterial meningitis (0.67 µg/l).

## Discussion

This explorative study is the first to provide plasma levels of the brain specific astroglial protein GFAP in patients with a broad spectrum of neurological diseases. Determined by a technically advanced GFAP prototype immunoassay, [4] we found low to very low GFAP blood values in the vast majority of patients, although many of them presumably had persistent neurological symptoms at the time point of blood draw. Together with recently published studies in stroke, [2]–[4] TBI, [12]–[14] and brain tumors, [7], [10], [11] our study provides further insights into the pathophysiology of GFAP release in neurological disorders, which is discussed below in detail. Furthermore, our study was not able to identify a condition or disease that may constitute a significant “specificity gap” in a GFAP blood test applied to identify ICH in patients with symptoms of acute stroke [4].

Our data support the assumption that (acute) structural disintegration of astroglial cells (i.e., mechanical disruption and/or necrotic cell death) and leakage through the blood brain barrier, allowing the protein to trespass from the intracellular compartment into the blood stream, [1] is a prerequisite for detectable GFAP release in peripheral blood. This convincingly explains the finding of massively elevated GFAP blood values, within minutes after symptom onset in ICH and traumatic brain injury, respectively. In ICH, a strong correlation exists between ICH volume and GFAP plasma values, further underlining the above-mentioned “mechanic” hypothesis. [3], [4] In the BE FAST trial, GFAP values of more than 100 µg/l were found in acute ICH patients with a mean of 29 µg/l, which is vastly higher than even the patients with the highest GFAP values in the present study. [4] In traumatic brain injury, the magnitude of GFAP elevation is strongly correlated to clinical severity and extent of the brain lesion. Values>7 µg/l were associated with an unfavorable outcome, while values>15 µg/l were not survived. [12]–[14] Three studies in the literature point to increased GFAP levels in plasma of patients with malignant glioma (glioblastoma) but not in metastasis, and suggest a potential use of this marker in the differential diagnosis of patients with newly identified cerebral mass lesions. [10], [11], [16] Malignant glioma tend to produce large amounts of GFAP, and many of them show substantial areas of necrotic cell death. Furthermore, it is well known that the blood brain barrier is disintegrated within the tumor. Thus, a gradual release of GFAP in to the blood stream appears plausible in glioblastoma, in contrast to other brain tumors such as metastases.

Apart from what was already known (i.e., elevated GFAP values in ICH, traumatic brain injury, and glioblastoma), we were not able to identify a disease or a group of diseases in our widespread explorative study that was consistently associated with increased blood GFAP levels. However, a few individual values from different disease categories were found clearly elevated and need further consideration: One patient had a subarachnoid hemorrhage with a concomitant parenchymal bleeding. [17] This explains the GFAP increase similar to what happens in *intracerebral* hemorrhage. All other patients with subarachnoid hemorrhage (n = 8), however, did not show elevated GFAP blood levels, suggesting that blood that enters the subarachnoid space not necessarily leads to brain parenchyma destruction. In one other patient from our series with elevated GFAP due to severe bacterial meningitis, we assume astroglial destruction caused by a concomitant infection of the brain parenchyma itself, which is a frequent finding in bacterial meningitis, or due to infectious vasculitis with cerebral ischemia. In contrast, three more patients with bacterial meningitis and basically all patients with other forms of CNS infection did not show increased GFAP blood values. Unfortunately, we were not able to provide an explanation for the elevated GFAP value in a patient with Parkinson’s disease. N = 13 other Parkinson patients and 10 more patients with movement disorders did not reveal increased GFAP value. The same was true for other neurodegenerative diseases, in particular Alzheimer’s disease. As there was no other concomitant disease that may explain GFAP release in this patient, we consider this value as a statistical outlier. It was reported that neuromyelitis optica (NMO), an autoimmune CNS disease targeting sites of high aquaporin-4 density in the spinal cord and the optic nerve, is associated with a marked loss of astrocytes. Increased GFAP levels were described in NMO patients, and it was suggested that this marker might help to differentiate the NMO-spectrum disease patients from patients with multiple sclerosis. [15] In our small series of four NMO patients and seventy-five multiple sclerosis patients, however, we found low GFAP blood values in both NMO- and multiple sclerosis patients not allowing for discrimination of both entities.

Regarding the potential application of GFAP as a biomarker of acute ICH, it is of importance to set the results of the present study into the context of the BE FAST trial, the largest study providing data on diagnostic accuracy of GFAP for identifying ICH in patients with symptoms of acute stroke so far. [4] In acute ICH patients, even at very early time points, GFAP plasma values as high as 100 µg/l were found, resulting from the immediate destruction of astroglial cells by the expanding hematoma. In contrast, in case of IS, GFAP release occurs in a more delayed manner, following the delayed dynamics of necrosis and cytolysis. [1] Among more than 160 patients with IS, only a few were found with (slightly) increased plasma concentrations, but still below 1 µg/l. 0.29 µg/l emerged as an optimal cut point to differentiate between ICH and IS. [4] Regarding potential “specificity gaps” of a future GFAP test applied to identify ICH, we may first consider typical “stroke mimicking” conditions. According to our study, typical stroke mimics such as migraine and epilepsy should not lead to false positive testing. However, a malignant brain tumor – which may sporadically present as a stroke mimic - may be falsely identified as an ICH. If the medical history of the patient is suggestive of a rather chronic than acute disease, the results of the test may be considered with caution. Difficulties may also occur if a patient with IS suffers from traumatic brain injury, e.g. as a consequence of a fall at symptom onset. In order to avoid false positive results, patients with an apparent trauma may not be tested. Most patients with subarachnoid hemorrhage do not present with a typical stroke syndrome. However, patients with parenchymal involvement may demonstrate focal neurological deficits, but are then likely to present with increased GFAP values, and will be correctly classified as “ICH”. All other diseases that may sporadically lead to increased GFAP values, e.g. severe bacterial meningitis, can hardly be considered as a relevant “stroke mimic”.

Taken together, GFAP is an attractive candidate protein that fulfills many of the criteria commonly asked for an “ideal” biomarker. It is abundantly present in the target organ, highly brain specific, biochemically stable and robust, and its plasma values are directly related to the extent of brain damage if used as an indicator of ICH. In this study, we were able to show, that age, sex, co-morbidities, risk factors as well as the vast majority of neurological diseases, especially the classical “stroke mimics” do not critically interfere with the use of GFAP as an ICH biomarker. Thus, bearing the above-mentioned limitations in mind, GFAP might be highly valid and reliable in indicating acute ICH in patients with symptoms suspicious of acute stroke.

## References

[1] Brunkhorst R, Pfeilschifter W, Foerch C (2010) Astroglial Proteins as Diagnostic Markers of Acute Intracerebral Hemorrhage-Pathophysiological Background and Clinical Findings. Translational Stroke Research. 1(4): 246–51. 10.1007/s12975-010-0040-6 24323552

[2] Dvorak F, Haberer I, Sitzer M, Foerch C (2009) Characterisation of the diagnostic window of serum glial fibrillary acidic protein for the differentiation of intracerebral haemorrhage and ischaemic stroke. Cerebrovasc Dis. 27(1): 37–41. 10.1159/000172632 19018136

[3] Foerch C, Curdt I, Yan B, Dvorak F, Hermans M, et al (2006) Serum glial fibrillary acidic protein as a biomarker for intracerebral haemorrhage in patients with acute stroke. J Neurol Neurosurg Psychiatry. 77(2): 181–4. 10.1136/jnnp.2005.074823 PMC2077601 16174653

[4] Foerch C, Niessner M, Back T, Bauerle M, De Marchis GM, et al (2012) Diagnostic accuracy of plasma glial fibrillary acidic protein for differentiating intracerebral hemorrhage and cerebral ischemia in patients with symptoms of acute stroke. Clin Chem. 58(1): 237–45. 10.1373/clinchem.2011.172676 22125303

[5] Eng LF, Ghirnikar RS, Lee YL (2000) Glial fibrillary acidic protein: GFAP-thirty-one years (1969–2000). Neurochem Res. 25(9–10): 1439–51. 10.1023/a:1007677003387 11059815

[6] Middeldorp J, Hol EM (2011) GFAP in health and disease. Progress in Neurobiology. 93(3): 421–43. 10.1016/j.pneurobio.2011.01.005 21219963

[7] Jung CS, Foerch C, Schanzer A, Heck A, Plate KH, et al (2007) Serum GFAP is a diagnostic marker for glioblastoma multiforme. Brain. 130(Pt 12): 3336–41. 10.1093/brain/awm263 17998256

[8] Missler U, Wiesmann M, Wittmann G, Magerkurth O, Hagenstrom H (1999) Measurement of glial fibrillary acidic protein in human blood: analytical method and preliminary clinical results. Clin Chem. 45(1): 138–41. 9895354

[9] Sofroniew MV (2009) Molecular dissection of reactive astrogliosis and glial scar formation. Trends in Neurosciences. 32(12): 638–47. 10.1016/j.tins.2009.08.002 PMC2787735 19782411

[10] Brommeland T, Rosengren L, Fridlund S, Hennig R, Isaksen V (2007) Serum levels of glial fibrillary acidic protein correlate to tumour volume of high-grade gliomas. Acta Neurol Scand. 116(6): 380–4. 10.1111/j.1600-0404.2007.00889.x 17986096

[11] Ilhan-Mutlu A, Wagner L, Widhalm G, Wohrer A, Bartsch S, et al (2013) Exploratory investigation of eight circulating plasma markers in brain tumor patients. Neurosurg Rev. 36(1): 45–56 doi: 10.1007/s10143-012-0401-6. Epub 2012 Jul 5 10.1007/s10143-012-0401-6 22763625

[12] Nylen K, Ost M, Csajbok LZ, Nilsson I, Blennow K, et al (2006) Increased serum-GFAP in patients with severe traumatic brain injury is related to outcome. J Neurol Sci. 15 240(1–2): 85–91. 10.1016/j.jns.2005.09.007 16266720

[13] Pelinka LE, Kroepfl A, Schmidhammer R, Krenn M, Buchinger W, et al (2004) Glial fibrillary acidic protein in serum after traumatic brain injury and multiple trauma. J Trauma. 57(5): 1006–12. 10.1097/01.ta.0000108998.48026.c3 15580024

[14] Yates D (2011) Traumatic brain injury: Serum levels of GFAP and S100B predict outcomes in TBI. Nat Rev Neurol. 2011 Feb 7(2): 63. 10.1038/nrneurol.2010.207 21391320

[15] Storoni M, Petzold A, Plant GT (2011) The use of serum glial fibrillary acidic protein measurements in the diagnosis of neuromyelitis optica spectrum optic neuritis. PLoS One. 6(8): e23489. 10.1371/journal.pone.0023489 PMC3158082 21876753

[16] Jung CS, Foerch C, Schanzer A, Heck A, Plate KH, et al (2007) Serum GFAP is a diagnostic marker for glioblastoma multiforme. Brain. 130(Pt 12): 3336–41. 10.1093/brain/awm263 17998256

[17] Nylen K, Csajbok LZ, Ost M, Rashid A, Blennow K, et al (2007) Serum glial fibrillary acidic protein is related to focal brain injury and outcome after aneurysmal subarachnoid hemorrhage. Stroke. 38(5): 1489–94. 10.1161/STROKEAHA.106.478362 17395862

